# Evaluation of a deep learning segmentation tool to help detect spinal cord lesions from combined T2 and STIR acquisitions in people with multiple sclerosis

**DOI:** 10.1007/s00330-025-11541-0

**Published:** 2025-04-04

**Authors:** Baptiste Lodé, Burhan Rashid Hussein, Cédric Meurée, Ricky Walsh, Malo Gaubert, Nicolas Lassalle, Guilhem Courbon, Agathe Martin, Jeanne Le Bars, Françoise Durand-Dubief, Bertrand Bourre, Adil Maarouf, Olivier Outteryck, Clément Mehier, Alexandre Poulin, Camille Cathelineau, Jeremy Hong, Guillaume Criton, Sophie Motillon-Alonso, Augustin Lecler, Frédérique Charbonneau, Loïc Duron, Alexandre Bani-Sadr, Céline Delpierre, Jean-Christophe Ferré, Gilles Edan, François Cotton, Romain Casey, Francesca Galassi, Benoit Combès, Anne Kerbrat

**Affiliations:** 1 Department of Neuroradiology, Rennes University Hospital, Rennes, France; 2 EMPENN Research Team, IRISA, CNRS‑INSERM‑INRIA, Rennes University, Rennes, France; 3 Department of Radiology, Rennes University Hospital, Rennes, France; 4 Neurology Department, Rennes University Hospital, Rennes, France; 5 Neurology A, Hôpital Neurologique Pierre Wertheimer, Hospices Civils de Lyon, University Lyon 1, Lyon, France; 6 Creatis LRMN, CNRS UMR 5220, Inserm U630, Université Claude Bernard Lyon 1, Lyon, France; 7 CHU Rouen, Department of Neurology, Rouen, France; 8 CRMBM, CNRS, Aix-Marseille Université, Marseille, France/APHM Hôpital de la Timone, Marseille, France; 9 University of Lille, INSERM, Department of Neuroradiology, CHU Lille, Lille, France; 10 Department of Radiology, Lyon University Hospital, Lyon, France; 11 CREATIS - CNRS UMR 5220 & INSERM U1044, University Claude Bernard Lyon 1, Lyon, France; 12 Department of Neuroradiology, Foundation Adolphe de Rothschild Hospital, Paris, France; 13 Paris Cité University, Paris, France; 14 Department of Neuroradiology, East Group Hospital, Hospices Civils de Lyon, Bron, France; 15 Department of Neuroradiology, Rouen University Hospital, Rouen, France; 16 CIC-P 1414 INSERM, University Hospital of Rennes, Rennes, France; 17 Department of Radiology, Lyon Sud Hospital, Hospices Civils de Lyon, Lyon, France; 18 Univ Lyon, Université Claude Bernard Lyon 1, Hospices Civils de Lyon, Fondation EDMUS, OFSEP, Centre de Recherche en Neurosciences de Lyon, Lyon, France

**Keywords:** Spinal cord, MRI, Multiple sclerosis, Deep learning-based tool, STIR sequence

## Abstract

**Objective:**

To develop a deep learning (DL) model for the detection of spinal cord (SC) multiple sclerosis (MS) lesions from both sagittal T2 and short tau inversion recovery (STIR) sequences and to investigate whether such a model could improve the performance of clinicians in detecting SC lesions.

**Materials and methods:**

A DL tool was developed based on SC sagittal T2 and STIR acquisitions from the imaging database of the French MS registry (OFSEP), including retrospective data from 40 different scanners. A multi-reader study based on retrospective data was performed between December 2023 and June 2024 to compare the performance of 20 clinicians in interpreting upper and lower SC acquisitions with and without the use of the tool. A ground truth was established by three experts. Sensitivity, precision, and inter-reader variability were evaluated.

**Results:**

We included 50 patients (39 females, median age: 41 years [range: 15–67]) with SC MRI acquired between February 2017 and December 2022. When reading with the tool, the clinicians’ mean sensitivity to detect SC lesions improved (from 74.3% [95% CI = 67.8–80.6%] to 79.2% [95% CI: 73.5–85.0%]; *p* < 0.0001), with no evidence of difference in the mean precision: (69.0% [95% CI: 62.8–75.2%] vs 70.1% [95% CI: 64.3–75.9%]; *p* = 0.08). Inter-reader variability in lesion detection was slightly improved with the tool (Light’s kappa = 0.55 vs 0.60), but without statistical difference (*p* = 0.056).

**Conclusion:**

The use of an automatic tool can help clinicians detect SC lesions in pwMS by increasing their sensitivity.

**Key Points:**

***Question*** *No tool to help detect MS SC lesions is used in clinical practice despite their frequency and prognostic value*.

***Findings*** *This DL-based tool led to improvement in clinicians’ sensitivity in detecting SC lesions from both sagittal T2 and STIR sequences, without decreasing precision*.

***Clinical relevance*** *Our study indicated the potential of a DL-based tool to assist clinicians in the challenging task of detecting SC lesions in people with MS on a combination of sequences commonly acquired in clinical practice*.

**Graphical Abstract:**

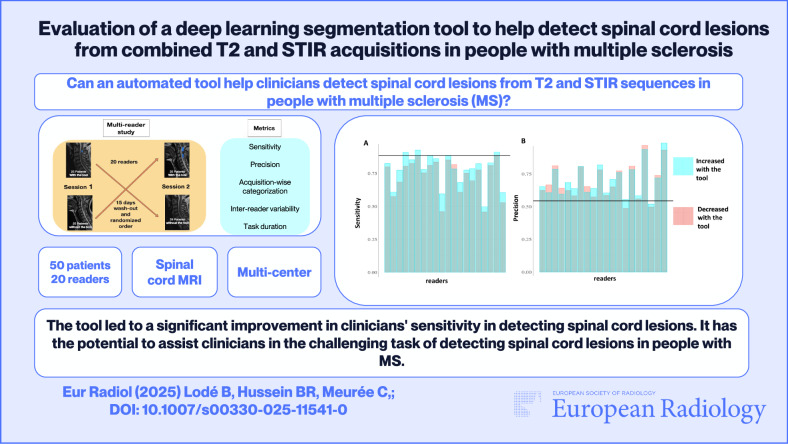

## Introduction

Spinal cord (SC) lesions are seen in up to 80% of people with multiple sclerosis (pwMS) [1], are included in diagnosis criteria [2], and have a strong prognosis value [3]. SC MRI is therefore performed very systematically during the initial assessment of the disease, although its interpretation is more challenging than that of brain MRI due to difficulties in acquiring images (e.g., small tissue size, pulsation artifacts). In view of the artifact risks, the international recommendations suggest acquiring at least two SC MRI sequences in pwMS from T2-weighted sequences, proton density-weighted sequences, or short tau inversion recovery (STIR) [4].

Deep-learning models have been widely applied to brain MRI analyses in pwMS [5, 6]. Some enable MS lesions to be segmented on a given brain MRI [7], others are dedicated to differentiating between MS lesions and non-specific white matter lesions [8], while others enable the detection of new MS lesions between two longitudinal brain MRI scans [9, 10]. The potential added value of similar tools for the analysis of SC MRI remains largely unexplored. Deep learning (DL)-based tools that segment SC lesions have been developed in a research context and have been included in the open-source SC toolbox [11, 12], but they have not been evaluated as an aid to SC lesion detection for radiologists in a clinical context. A diagnostic tool to differentiate between tumoral and inflammatory SC lesions has recently been proposed [13]. At present, no tool to help detect SC lesions in pwMS is used in clinical practice by radiologists. Moreover, no tool combining the analysis of two SC sequences, as recommended in clinical routines, has been proposed and evaluated.

In this study, our main objective is to assess the impact of a segmentation tool as an aid to detect SC MS lesions from both T2 and STIR, on clinician’s sensitivity and precision. Our secondary objectives were to evaluate its impact on interpretation time and inter-reader variability.

## Materials and methods

We developed a DL tool to detect SC MS lesions, and we performed a multi-reader, multi-case retrospective study with a fully crossed design. The study was approved by the local ethics advisory committee (no. 24.100). All data were extracted (de-identified) from the French MS registry in October 2023 (www.ofsep.org) and included clinical and imaging data from 38 expert MS centers collected during patients’ routine follow-up visits [14, 15] (NCT02889965).

### Characteristics of the segmentation model

Details about the segmentation model are provided in the Supplemental Material. Briefly, it is based on a five-level U-net architecture [16] with one input channel for each of the two sequences of interest and one single output channel. The model was trained with a combined dice and cross-entropy loss function (deep supervision was applied) on an annotated dataset of 140 cases from 40 scanners and with a diverse range of lesion presentations extracted from the OFSEP database. The model output post-processing was optimized on a dataset of 21 cases for the task assessed in our work (favoring a good sensitivity with the risk of deteriorated precision). The resulting model achieved a lesion-wise sensitivity of 0.89 and a precision of 0.64 on a test set of 40 cases.

### The multi-reader study design

**Fig. 1:**
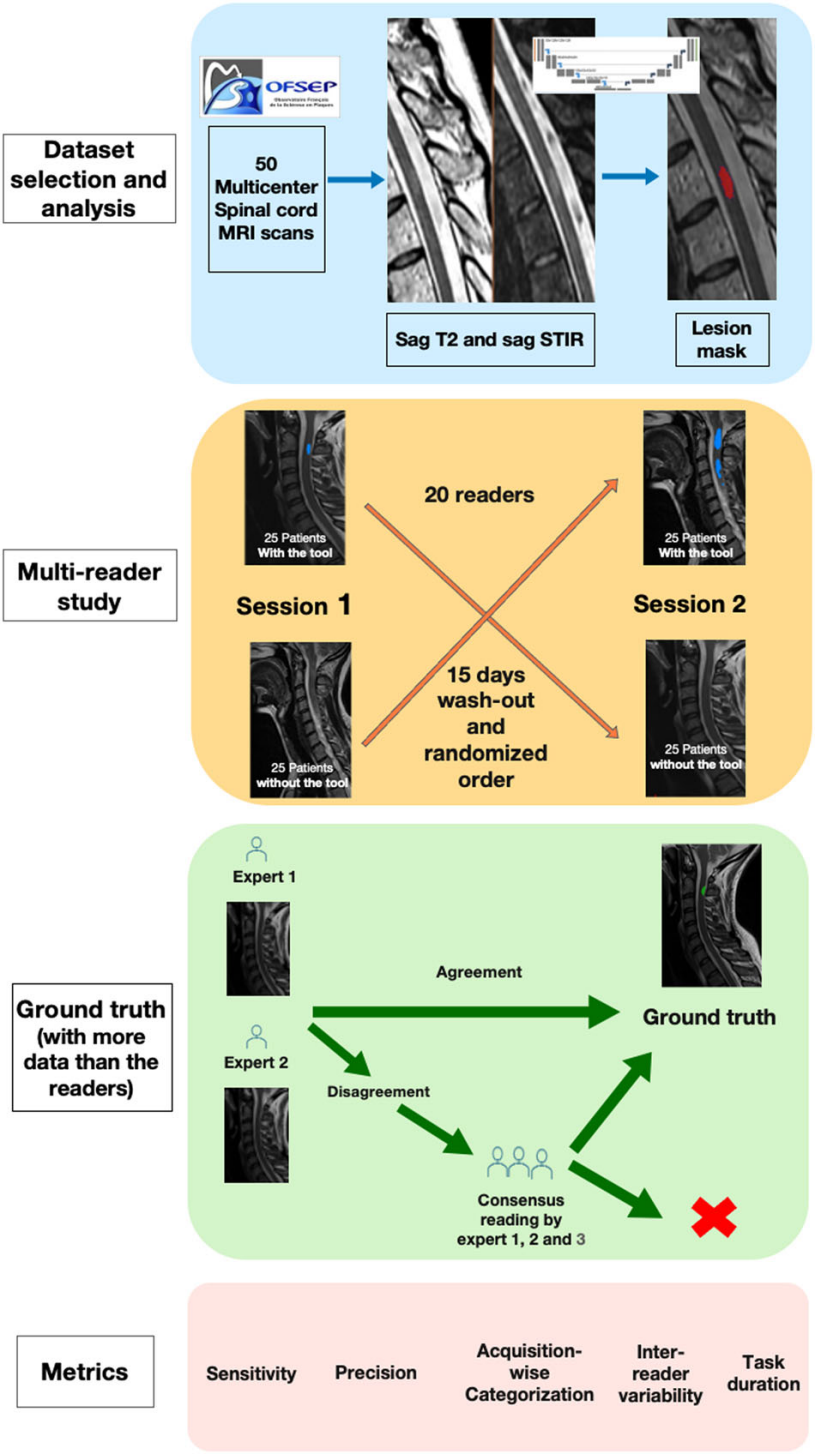
Summary of the multi-reader study design

The multi-reader study was conducted between December 2023 and June 2024 (Fig. 1). Twenty clinicians annotated cervical and thoracic SC sagittal T2 acquisitions from 50 pwMS, with the systematic help of sagittal STIR. A web-based annotation tool resembling a standard picture archiving and communication system was used to annotate lesions by clicking once on each lesion (Supplementary Fig. 1). Each reader underwent a standardized training protocol, including a 20-min presentation and a written tutorial. A timer recorded the time spent on each MRI volume. Each patient was analyzed twice by each reader, with and without the tool, in a randomized order across two sessions at least 15 days apart. At the end of the experiment, we asked each reader to complete a questionnaire concerning their potential expectations of a tool to assist in the detection of SC MS lesions in clinical practice. Each item was scored from 1 to 5, ranging from “completely disagree” to “completely agree”.

### Dataset

SC MRI acquisitions were extracted from the OFSEP database. Inclusion criteria were images acquired from 2017, with both sagittal T2 and STIR acquisitions available at both upper and lower SC levels, with at least another SC MRI timepoint available. Non-inclusion criteria were patients who were already included in the training dataset. Exclusion criteria were a pathologic condition of the SC other than MS and poor imaging quality. Initially, 55 patients were selected, five of whom were excluded due to poor imaging quality, resulting in a final cohort of 50 patients.

### Readers

Radiologists and neurologists with varying levels of expertise and who were not involved in the tool development process were recruited for the experiment. Senior neuroradiologists and neurologists were recruited at the meeting of the OFSEP imaging group and came from French MS expert centers. Junior radiologists and neurologists were residents from the same centers. The general radiologists had no particular specialization in the follow-up of MS patients.

### Ground truth

Ground truth segmentation was established independently by a neurologist with 10 years of experience in MS SC MRI reading (A.K.) and a radiology resident (B.L.) using ITK-SNAP software (version 4.0.1). They had more data at their disposal than the readers (SC MRI data on at least one other time-point and axial acquisitions). Discrepancies between them were resolved through adjudication with a third expert, a neuroradiologist with six years of experience (N.L.).

Secondarily, the 20 readers’ annotations were grouped together on each MRI volume and were independently reviewed by B.L. and A.K. to construct a revised ground truth to potentially reintegrate lesions that would have been missed in the initial ground truth. We considered that a reader had correctly identified a lesion if he had clicked on any voxel within the ground truth lesion mask.

### Statistical analysis

Statistical analyses were performed using R (version 4.2.2).

#### Primary outcome

to assess a difference in mean readers’ sensitivity and/or precision to detect SC lesions with or without the tool. Prior to the study, we established through simulation that, under simplifying assumptions and for a Type I error of 0.05, a Type II error of 0.20, and an improvement in sensitivity of 10% with the tool (based on an average performance of experts alone of 0.60), a set of 50 patients with at least seven experts was sufficient. The individual mean sensitivity together with its 95% confidence interval (CI) was computed for each condition, with or without the tool. The overall mean sensitivity was tested for equality between the two conditions using a regression including a condition, acquisition, and a reader-fixed effect. The same analysis was conducted for the mean precision.

#### Secondary outcomes

We studied supplemental metrics as an exploratory analysis. Corresponding *p*-values associated with absences of effect are provided without correction for multiple comparisons.

### Supplemental assessment of difference in mean sensitivity and precision

The main analysis was repeated according to readers’ experiences (< 10 and ≥ 10 years), readers’ specialty (neurologists with expertise in MS, radiologists, and neuroradiologists), acquisitions coverage (upper and lower SC), MRI magnetic field, slice thickness, and image in-plane resolution. Then, sensitivity and precision were computed for the model alone and were compared to the performances of the reader alone using a linear model with an acquisition and a condition (reader without the tool vs model alone) fixed effect.

### Lesion count and lesion-wise sensitivity and precision

The average number of true positive (TP) lesions detected per reader was computed for each condition. This number was tested for equality between the two conditions using a paired *t-*test. Lesion-wise sensitivity and precision were analyzed aggregating all lesions from all scans. The individual lesion-wise sensitivity, together with its 95% CI, was computed for each condition and was then tested for equality between conditions using a logistic regression including a condition and an acquisition fixed effect. The same analysis was reported for the lesion-wise precision. These analyses were repeated according to lesion volume.

### Acquisition-wise categorization

Each acquisition was categorized in three classes: no lesion, 1 or 2 lesions, and ≥ 3 lesions. The overall accuracy associated with this categorization was computed for the two conditions and tested for equality using a logistic regression including a condition, a patient, and a reader effect.

### Task duration

The mean time elapsed for each acquisition, each reader, and each condition was estimated after having filtered out the 5% upper values. This was done to avoid outliers due to delay during the task as a result of external solicitations and tested for equality between conditions using a Wilcoxon rank sum test.

### Inter-reader variability

The inter-reader differences in detected lesions within each condition were reported as multi-rater Light’s kappa. The pooled inter-reader standard deviation associated with the number of lesions detected in each condition was computed and compared using a linear model with a condition and an acquisition fixed effect.

### Revised ground truth

The main analyses were repeated with the revised ground truth.

## Results

### Patients, MRI acquisitions, and readers’ characteristics

We included 50 patients (39 females, 11 males; 44 relapsing, 6 progressive MS) with SC MRI acquired between February 2017 and December 2022. The median age was 41 years (range: 15–67 years) and the median Expanded Disability Status Scale score was 2 (range: 0–6.5). The data come from Siemens (46), Philips (2), and GE (2) scanners (details in Supplementary Table 1). Thirty-eight datasets came from 1.5-T scanners and 12 from 3-T scanners. The median in-plane resolution was 0.42 × 0.42 mm^2^ (range: 0.34–0.86) for the sagittal T2 and 0.47 × 0.47 mm^2^ (range: 0.42–1.00) for the sagittal STIR. The median slice thickness was 3 mm (range: 2.00–3.85) for the sagittal T2 and 3 mm (range: 2.50–3.85) for the sagittal STIR. The readers’ characteristics are summarized in Table 1.

**Table 1:** Readers’ characteristics

	All readers *n* = 20	Physicians category		
		Neuro-radiologists *n* = 8	Radiologists *n* = 5	Neurologists experienced in MS *n* = 7
Number of years of experience (median, range)	8 (0–43)	12 (2–20)	2 (2–3)	12 (0–43)
Number of SC MRI of pwMS read per month (median, range**)**	9 (0–100)	35 (5–50)	1 (0–2)	10 (2–100)

*pwMS* people with MS

### Ground truth characteristics

A total of 170 lesions were detected by both experts 1 and 2. They disagreed on 46 lesions. Of these, 36 were validated during the consensual reading with the third expert, resulting in a total number of 206 lesions included in the ground truth (148 in upper SC and 58 in lower SC).

### Acquisition-wise sensitivity and precision

**Fig. 2:**
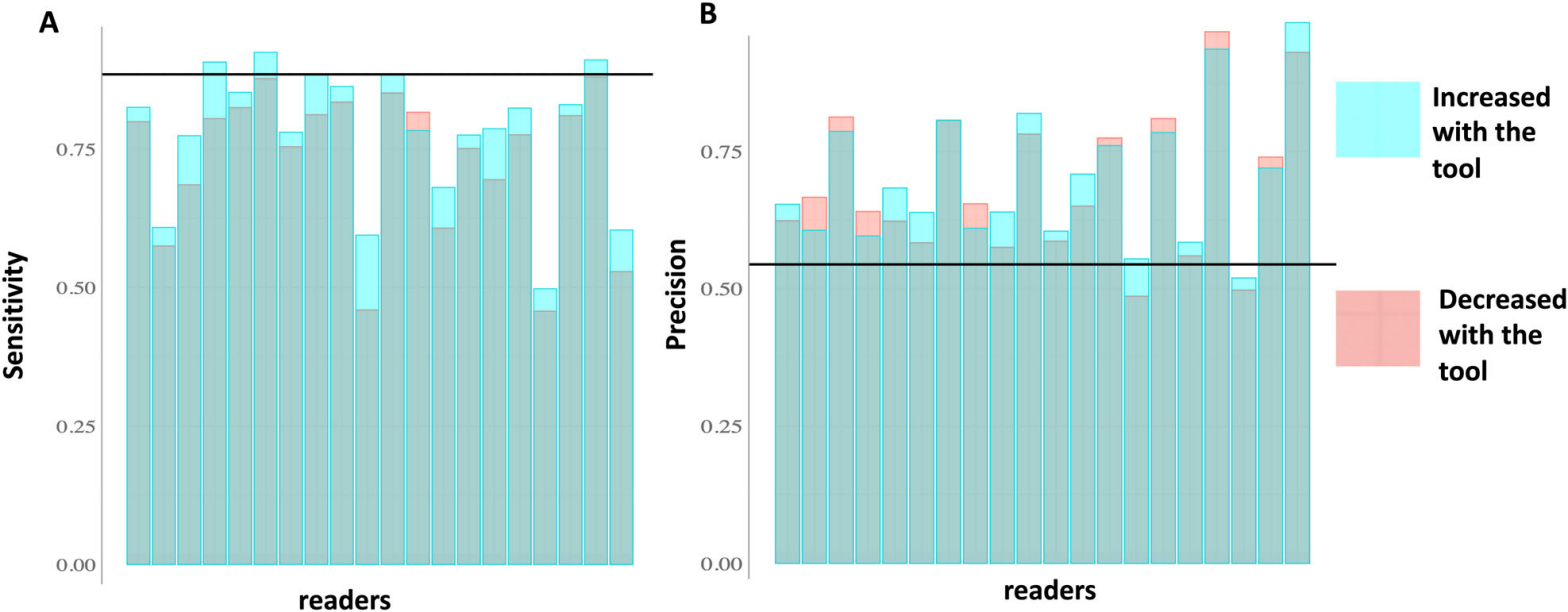
Lesion detection performance for each reader and each session: **A** for sensitivity and **B** for precision. The individual performance of each reader is represented by a histogram, where light blue bars on top indicate performance augmented with the tool and red bars on top indicate performance lowered with the tool. The black line on each graph indicates the performance of the tool alone

**Fig. 3:**
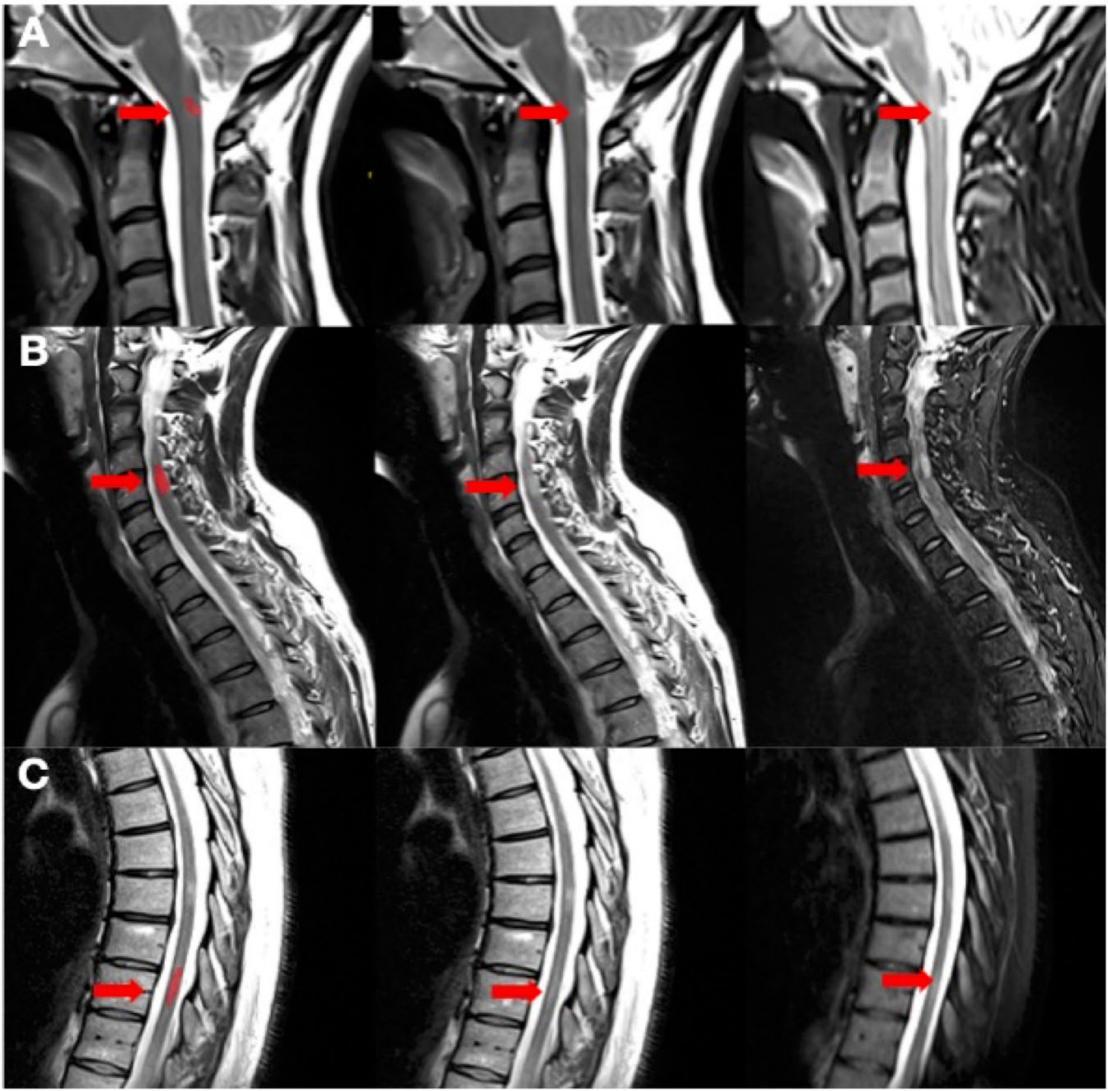
Examples of lesions that were detected more frequently by readers with the tool than without the tool. The first column, is sagittal T2 acquisition with the lesion of interest mask in red; the second column, is sagittal T2 acquisition; the third column, is sagittal STIR acquisition. The lesions of interest are localized by the red arrow. Lesion **A** was detected by eight more readers with the tool than without the tool, lesion **B** by seven, and lesion **C** by six

The sensitivity of the readers was significantly improved with the tool (79.2% [95% CI: 73.5–85.0%] vs 74.3% [95% CI: 67.8–80.6%]; *p* < 0.0001), with no evidence of a difference in precision (70.1% [95% CI: 64.3–75.9%] vs 69.0% [95% CI: 62.8–75.2; *p* = 0.08) (Table 2 and Figs. 2 and  3). For sensitivity, we observed similar results when grouping readers according to their experience or specialty. Regarding precision, we only found a significant improvement with the tool in readers with less than 10 years of experience (*p* = 0.032).

**Table 2:** Acquisition-wise sensitivity and precision for all readers and for different subgroups, without the tool or with the tool (expressed in %, with 95% CIs)

	Sensitivity			Precision		
	Without the tool	With the tool	*p* value	Without the tool	With the tool	*p* value
All readers						
*n* = 20	74.3 (67.8–80.6)	79.2 (73.5-85.0)	< 0.0001	69.0 (62.8–75.2)	70.1 (64.3– 75.9)	0.082
According to the reader’s experience						
< 10 years *n* = 11	74.1 (64.7–83.6)	78.2 (69.8–86.6)	0.006	69.3 (58.9–79.8)	72.0 (62.5–81.4)	0.032
≥ 10 years *n* = 9	74.4 (63.6–85.2)	80.5 (70.8–90.1)	0.0001	68.7 (60.3–77.0)	67.8 (59.9–75.6)	0.687
According to the reader’s expertise						
Neurologist *n* = 7	69.0 (45.9–91.0)	75.6 (63.8–85.7)	0.0005	69.7 (61.1–78.3)	69.2 (60.4–78.0)	0.492
Radiologist *n* = 5	68.5 (44.9–89.7)	73.1 (50.9–92.4)	0.053	79.1 (58.3–99.8)	81.3 (63.2–99.4)	0.512
Neuro-radiologist *n* = 8	82.5 (77.3–84.8)	86.2 (80.3–89.1)	0.004	62.2 (53.0–71.3)	63.8 (56.2–71.4)	0.094
According to the level of the SC						
Upper part *n* = 20	73.4 (67.3–79.5)	78.0 (72.0–84.0)	0.0007	70.1 (64.2–75.9)	70.8 (65.4–76.2)	0.2787
Lower part *n* = 20	75.6 (65.2–79.5)	81.3 (72.1–83.6)	0.002	67.9 (60.4.8–75.4)	69.3 (62.6-76.1)	0.1076

For the model alone, the mean acquisition-wise sensitivity and precision were 88.6% (83.8–93.1%) and 54.4% (46.0–62.8%), respectively. We observed a significant difference vs the reader alone in mean sensitivity (*p* < 0.0001) but not in mean precision (*p* = 0.14).

The impact of MRI magnetic field, slice thickness, and image in-plane resolution on tool and reader performance is reported in Table 3.

**Table 3:** Acquisition-wise sensitivity and precision according to MRI magnetic field, slice thickness, and in-plane resolution (expressed in %, with 95% CIs)

	Sensitivity				Precision			
	Tool alone	Readers without the tool	Readers with the tool	*p* value (readers with vs without the tool)	Tool alone	Readers without the tool	Readers with the tool	*p* value (readers with vs without the tool)
According to MRI magnetic field								
1.5 T *n* = 38	86.9 (79.5–94.2)	74.2 (66.7–81.7)	79.2 (73.1–85.4)	0.0006	51.8 (40.8–62.8)	68.1 (62.2–74.1)	70.1 (64.0–76.2)	0.11
3 T *n* = 12	90.9 (85.8–96.0)	74.3 (68.8–79.8)	79.2 (73.6–84.8)	0.002	58.2 (44.6–71.8)	74.3 (63.5–77.0)	70.3 (64.3–76.3)	0.35
According to slice thickness for sagittal T2								
≤ 3 mm *n* = 31	89.2 (84.2–94.2)	72.3 (65.7–78.8)	77.4 (61.7–83.1)	0.00008	56.2 (45.5–66.8)	70.6 (56.2–68.2)	71.3 (65.5–77.0)	0.65
> 3 mm *n* = 19	87.5 (77.6–97.4)	77.6 (70.8–84.3)	82.3 (76.1–88.4)	0.01	51.3 (36.9–65.7)	66.6 (59.5–73.6)	68.1 (64.6–76.6)	0.02
According to in plane resolution for saggital T2								
≤ 0.5 × 0.5 mm^2^ *n* = 29	89.5 (84.4–94.5)	71.3 (64.6–77.9)	76.9 (70.8–82.8)	0.00008	53.6 (43.3–63.8)	68.2 (61.9–74.5)	68.7 (62.7–74.6)	0.50
> 0.5 × 0.5 mm^2^ *n* = 21	87.0 (77.2–97.0)	79.3 (62.9–85.6)	83.2 (77.8–88.6)	0.01	55.6 (40.7–70.5)	70.7 (65.6–77.8)	72.5 (66.6–78.4)	0.02

### Lesion count and lesion-wise sensitivity and precision

The mean number of TP lesions detected per reader was 145 without the tool vs 152 with the tool (*p* = 0.0004), and the mean number of FP was 75 with the tool vs 74 without (*p* = 0.67). The lesion-wise sensitivity was improved with the tool (73.9% [95% CI: 68.0–79.8%] vs 70.7% [95% CI: 64.3–77.0%]; *p* = 0.0003) with no evidence of lesion-wise precision improvement (69.8% [95% CI: 64.3–75.3%] vs 69.1% [95% CI: 63.1–75.0%], *p* = 0.47). The model alone has a TP of 175 and a number of FP of 103, providing a lesion-wise sensitivity and precision of 85.4% and 62.9%.

The lesion-wise sensitivity and precision of the model alone were 79.5% and 50.8% for small lesions (< 100 mm^3^) and 89.3% and 94.9% for large lesions (≥ 100 mm^3^). The lesion-wise sensitivity of the readers was increased with the tool for both small (*p* = 0.00009) and large lesions (*p* = 0.039) (Supplementary Table 2).

### Acquisition-wise categorization

Based on the ground truth, each of the 100 MRI volumes was categorized as no lesion (*n* = 33), one or two lesions (*n* = 36), or ≥ 3 lesions (*n* = 31). Results of the categorization from the readers are summarized in Supplementary Table 3. We observed no evidence of a difference in categorization with or without the tool (*p* = 0.22).

### Task duration

The median time taken by readers per SC MRI volume was increased with the tool (64.9 s [IQR = 36.0–108.2] vs 58.1 s [IQR = 32.3–103.2] without; *p* = 0.001).

### Inter-reader variability

**Fig. 4:**
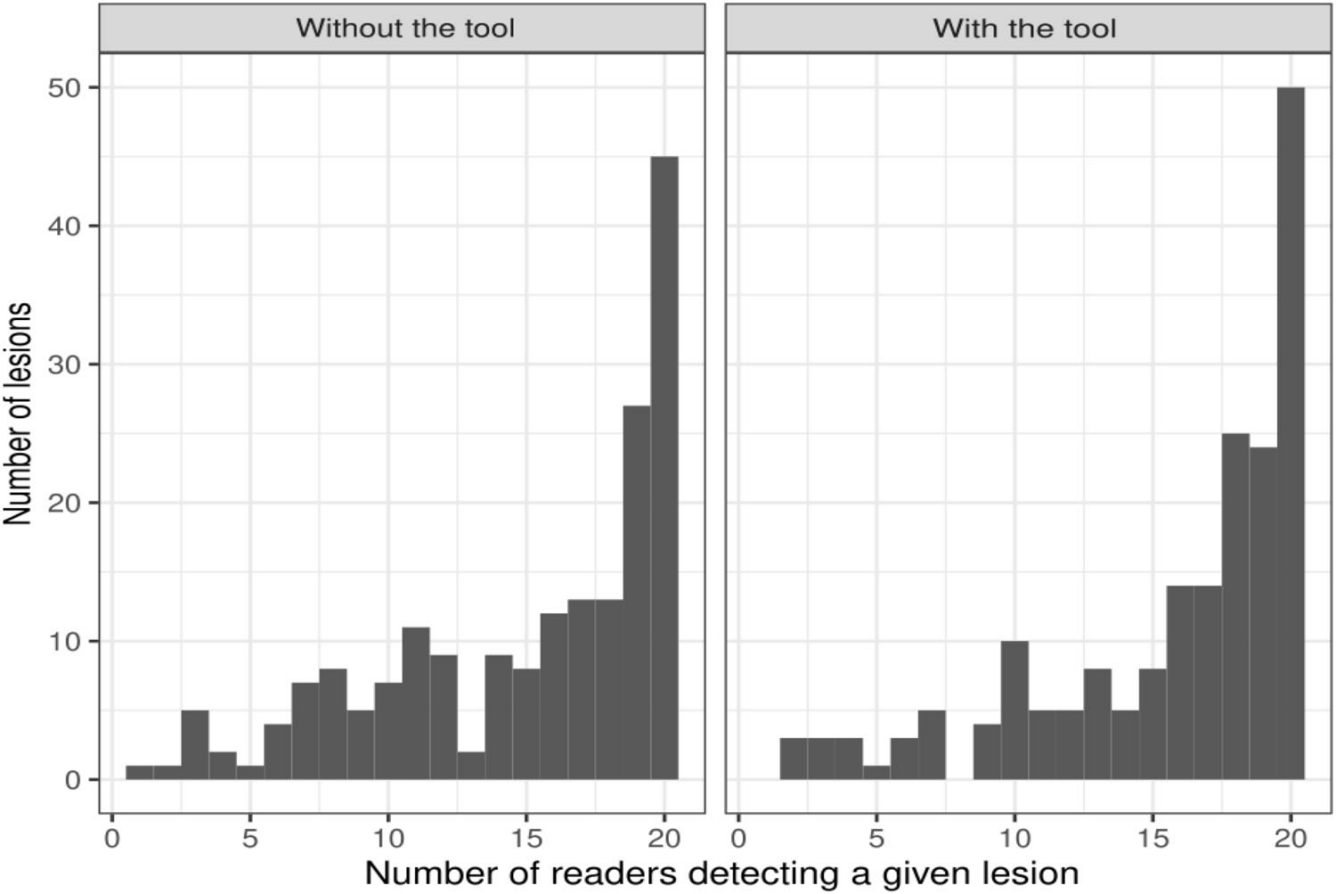
Number of experts having detected each lesion present in the ground truth without and with the tool. We observe a wide dispersion in the number of experts who discovered each lesion, which is slightly improved by using the tool

**Fig. 5:**
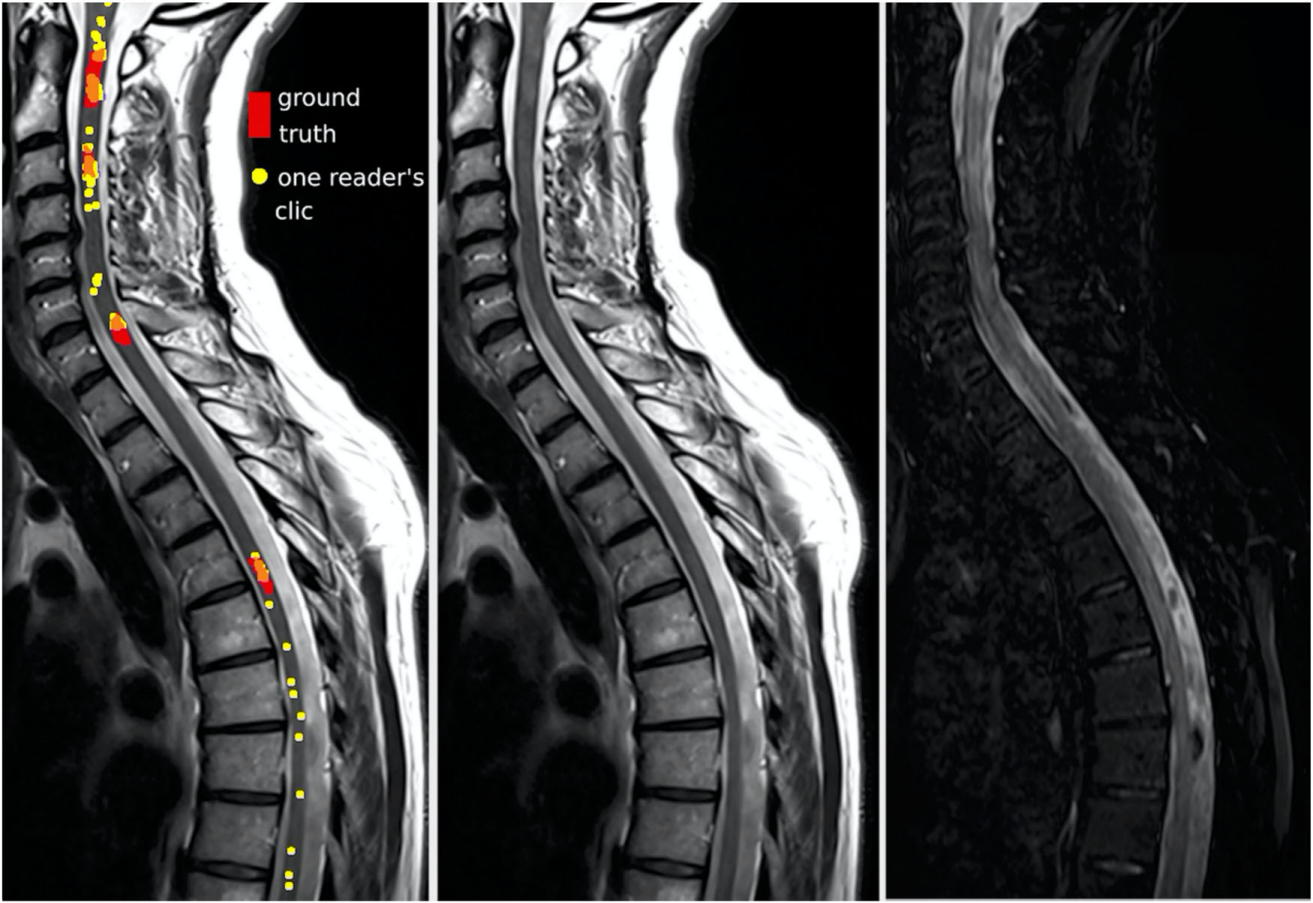
Example of inter-reader variability for the same SC MRI acquisition. Column 1: sagittal T2 acquisition with the ground truth mask in red, all the experts’ clicks on what they thought was a lesion in yellow, and the intersection of ground truth and expert clicks in orange. Column 2: sagittal T2 acquisition. Column 3: sagittal STIR acquisition

The Light’s kappa was 0.55 without the tool, indicating a moderate inter-expert agreement (Figs. 4 and 5), and was slightly increased at 0.60 with the tool. Similarly, the pooled standard deviation associated with the number of reported lesions was 1.54 without the tool and 1.39 with the tool (*p* = 0.056).

### Revised ground truth

Using revised ground truth, we observed a significant improvement in mean sensitivity with the tool (69.9% [95% CI = 63.8–76.0%] to 74.5% [95% CI = 68.9–80.1%]; *p* value < 0.0001) but no statistical difference in precision.

### Readers’ survey

The main results from the survey are reported in Table 4, indicating rather positive feedback on the use of AI for SC MRI reading.

**Table 4:** Readers’ survey, number of responses per item (out of 20 readers)

	Completely disagree	Rather disagree	Don’t know	Rather agree	Completely agree
“I am confident when I read an SC MRI”	0	6	1	13	0
“I think AI can be useful to help me read an SC MRI”	0	0	0	11	9
“I think the proposed system allows me more comfort in reading an SC MRI”	0	3	3	12	2
“I would like to use such a system in my clinical practice”	0	3	4	9	4
“I would advise such a system to a junior”	1	4	2	8	5
“I would advise such a system to a senior”	0	2	4	12	2

*SC* spinal cord

## Discussion

In this study, we developed and validated a DL tool to detect SC MS lesions on combined sagittal T2 and STIR acquisitions commonly acquired in clinical practice. We have demonstrated that the concurrent use of the tool led to significant improvement in readers’ sensitivity without lowering precision and tended to reduce inter-reader variability. The majority of clinicians also reported that using the tool improved their comfort when reading SC images.

To date, no study has evaluated the use by clinicians of an AI-based tool to help detect MS SC lesions. As illustrated by our survey, there is a need for this difficult task, which can have an impact on the diagnosis, the evaluation of the prognosis, and the treatment proposed to pwMS [1, 3, 17]. A precise count of the number and volume of SC lesions is not usually conducted in clinical practice, even though both the number and volume of MS SC lesions in a recent study were associated with future accumulation of disability progression independent of clinical relapses in pwMS [18]. The use of AI tools in clinical practice makes this precise monitoring realistic.

A previous study evaluated an AI model, segmenting MS SC lesions from sagittal T2w or axial T2*w, compared to manual segmentation [12]. They reported lesion-wise sensitivity at 83.3% and lesion-wise precision at 76.9% (vs 85.4% and 62.9% in our study, based on data acquired only in clinical practice). Although direct comparison with this method is beyond the scope of this study, it should be emphasized that our tool was deliberately optimized for high sensitivity—and, thus, lower precision—to improve the number of MS lesions detected by the readers, based on the assumption that they would remove FP. Our results confirm this hypothesis, with a significant improvement in clinicians’ sensitivity in SC lesion detection with the tool, without lowering precision. It should also be noted that this increase in clinician sensitivity with the tool is observed for both small and large lesions, and whatever the magnetic field of the acquisitions, the thickness of the slices, and the in-plane resolution. However, clinicians must be aware of the potential FP and exercise their critical judgment when interpreting the tool’s suggestions.

Another original aspect of our study is the development of a tool that takes into account two different major sequences to detect SC lesions. Indeed, as recommended by the latest recommendations [4], a single acquisition of a T2-weighted sequence is not sufficient, and a second sequence is required to confirm the presence of lesions and exclude artifacts [19–21]. While several combinations of sequences are proposed in the recommendations, we have chosen in this study to develop and test a model taking into account the sequences most commonly acquired in clinical practice in France (T2 and STIR). However, unlike brain imaging, in which a clear consensus exists (3D FLAIR), the acquisition combinations for the SC can be multiple, depending on the MRI scanner model and the habits. Recently, sequences such as phase-sensitive inversion recovery [22–24] or MP2RAGE [25] have demonstrated better detection, with better confidence, of cervical SC MS lesions compared to sagittal T2 and STIR, due to optimized contrast and high spatial resolution. The tool, therefore, could be adapted in the future to consider these different combinations of sequences.

The tool did not allow the readers to reduce their interpretation time. This result contrasts with the reduction in interpretation time of brain MRIs reported with tools to help detect new brain lesions [10]. However, the interpretation of SC MRIs is a particularly delicate task, and the addition or removal of the mask to validate or invalidate the lesion proposed by the tool probably explains this result. Furthermore, despite this extended interpretation time, it remains reasonably short (approximately 2 min per patient for an analysis of the full SC), and is accompanied by an increase in sensitivity and a better feeling of comfort when interpreting the images. Finally, this was the first time that readers had used the tool. Reading time could be reduced with practice.

### Limits and perspectives

Our study has several limitations. First, it is well documented that radiologists’ performance in interpreting examinations is different in a clinical context than in a retrospective research context [26]. Therefore, further experiments are needed to validate the model in routine use. Second, the task of detecting SC lesions is inherently variable and difficult, as illustrated by the moderate inter-reader agreement for SC MS lesion detection in our study, which also potentially affects the constitution of the ground truth by experts. To minimize this difficulty, the three experts involved had access to more data than the readers did, such as axial acquisitions and follow-up MRI scans. Moreover, the consistency of our findings with the exploratory analysis using the revised ground truth (from all the lesions detected by the 20 readers) suggests the robustness of our results. Thirdly, we did not ask the readers to segment each lesion, but we considered that they had correctly identified a lesion if they clicked on any voxel within the ground truth lesion mask. This methodological choice likely introduces a bias in the estimation of the clinicians’ lesion detection sensitivity and precision. Thus, if the reader clicks *n* times on a TP lesion, it is considered the same as if they click once, whereas if they click *n* times on a given false positive lesion, it is considered *n* false positives. Sensitivity may therefore be slightly overestimated in our study, while precision may be underestimated. However, the main objective of our study was to compare the mean readers’ sensitivity and precision in detecting SC lesions with or without the tool. This same bias related to our methodological choice was thus applied in both cases and probably had little impact on the main result of our study. Finally, the developed tool is designed to detect SC lesions in pwMS in cross-sectional MRI. It does not differentiate between SC MS lesions and differential diagnoses, such as an intramedullary tumor or a Neuromyelitis Optica Spectrum Disorder. Furthermore, a longitudinal tool, to assist new SC lesions detection between two MRI scans, remains to be developed and tested.

## Conclusion

Our multi-reader study demonstrated that the concurrent use of AI in SC MRI interpretation increases the sensitivity of radiologists and neurologists in detecting MS SC lesions, without reducing their precision, and improves their comfort in performing this difficult task. Further studies are needed to evaluate the potential of these tools with other sequence combinations, and as an aid to the detection of new SC lesions.

## Supplementary information

ELECTRONIC SUPPLEMENTARY MATERIAL

## Data Availability

The individual de-identified imaging data can be obtained upon request to the OFSEP Scientific Board (https://www.ofsep.org/en/data-access). The data generated and analyzed during the study are available from the corresponding author by request. The full study protocol is available at https://osf.io/2q6sb/.

## Abbreviations

DL: Deep learning
MS: Multiple sclerosis
PwMS: People with multiple sclerosis
SC: Spinal cord
STIR: Short tau inversion recovery
TP: True positive
