# Supplementary material for: Evaluation of a deep learning segmentation tool to help detect spinal cord lesions from combined T2 and STIR acquisitions in people with multiple sclerosis
Source: Eur Radiol. 2025 Apr 4;35(10):5954–64. doi: 10.1007/s00330-025-11541-0 (PMC12417267; doi:10.1007/s00330-025-11541-0)

Evaluation of a deep learning segmentation tool to help detect spinal cord lesions from combined T2 and STIR acquisitions in people with multiple sclerosis

ELECTRONIC SUPPLEMENTARY MATERIAL

Characteristics of the Segmentation Model

The developed deep-learning tool to segment MS lesions from a pair of T2w and STIR spinal cord images is based on a five level U-net with one input channel for each of the two sequences of interest and one single output channel trained on a heterogeneous set of 140 acquisitions from MS patients. Its main characteristics are detailed below.

Training, Validation and Testing datasets: The model was trained and tested on a dataset of spinal cord MRI scans extracted from the OFSEP database. Main inclusion criteria were i) pwMS with an OFSEP consentment to re-use clinical and imaging data signed; ii) with both sagittal T2 and T2 STIR acquisitions available at upper and/or lower spinal cord levels; iii) with at least another spinal cord MRI timepoint available, in order to improve lesion segmentation by annotators. Seven junior radiologists (in training) carried out an initial annotation of spinal cord lesions on T2w images with the systematic help of the STIR sequence, of previous acquisitions, and of other sequences when available (e.g. axial T2 or T2*). Each annotator underwent a standardized training protocol including a face to face presentation and a written tutorial. Each subject was annotated by one of the 7 radiologists, then a senior neurologist with 10 years of experience in spinal cord image analysis subsequently validated or corrected each annotation. Overall two hundred and ten acquisitions from 40 different MRI scanners were annotated. Nine were finally excluded due to poor-quality acquisitions. The final annotated dataset comprised 142 subjects with 201 acquisitions (134 cervical and 67 dorsal)

from various 1.5T (102 acquisitions) or 3T (99) scanners from three manufacturers (158 from Siemens, 31 from GE and 12 from Phillips). Then, the subjects were split into training (140 acquisitions), validation (21 acquisitions), and test (40 acquisitions) sets with stratified randomization to maintain similar distributions of lesion load and device manufacturers. The amount of acquisitions in each set was selected to favor the training set.

Data preprocessing: Before feeding the neural network, data were preprocessed and splitted into patches. More specifically, the data were first resampled to 0.5mm isotropic voxels. Second, the spinal cord was segmented on the T2w using the tool sct_deepseg_sc (De Leener et al. 2017). Then, the spinal cord centerline was extracted by taking the centre-of-mass of the spinal cord segmentation mask for each axial slice. In the few cases of failure to detect the spinal cord the manual approach using sct_get_centerline with the viewer method was used. Then T2w and STIR images were cropped to a cylindrical area with diameter 35mm around the T2w centreline. The T2w mask was used to crop both images because the images were generally sufficiently aligned and the spinal cord would still be captured in the STIR image. Strongly misaligned T2w/STIR were grossly manually rigidly registered (three cases overall). Then, the cropped STIR image was registered to the cropped T2w image using the deep learning-based nonlinear registration algorithm of SCT (sct_register_multimodal) (De Leener et al. 2017). In six cases, this registration failed completely or introduced significant artefacts and these images were excluded from the dataset (and not accounted for in the reported sample size). The T2w and STIR images were cropped again to the intersection of their FOV. The resulting images were finally processed into 48x48 voxel patches along the z-axis, effectively leading to a straightened spinal cord but without interpolation of intensity values and stacked into 3D patches of 48x48x384 (padded if needed).

Neural network architecture and training: The architecture used was a 3D U-Net with five levels with 32, 64, 128, 256, and 320 convolutional filters, strided convolutions to down-sample or up-sample at each level. Only the z-axis was down-sampled at the lowest level. The loss function was a combined Dice and cross-entropy loss, and a deep supervision loss was applied. An L2 penalization term was added (with decay factor) to the loss and drop-out (p=0.2) was applied along training. The model was trained for 2000 epochs, where an epoch consisted of seeing two randomly sampled (augmented) patches from each volume. At each epoch, data augmentation was applied to the images including random motion, gamma correction, random bias field, blur, random anisotropy, flipping, random noise, and random affine or elastic deformations. We used an SGD optimizer, learning rate of 0.01, L2 weight decay factor of 3×10-5, momentum factor of 0.99, a polynomial learning rate decay with power equal to 0.9 and a batch size of 8 (maximum allowed by available GPU RAM given the patch size and model architecture). Layers were initialized with random values from the uniform distribution U(-1/sqrt(n), 1/sqrt(n)), where n is the number of parameters in the layer. After training, the epoch that achieved the best loss on the validation set was selected as the final model.

Data postprocessing: The neural network softmax output maps were then postprocessed to generate binary segmentation masks in the frame of the original T2w volumes. For that purpose, a post-processing pipeline that mirrors the preprocessing one is applied to the probability maps generated by the model to adjust them to the corresponding input T2w. The post-processed probability maps were then binarised in order to obtain lesion masks specifically for this study. In particular, we aimed at proposing a model optimized for the mean lesion-wise sensitivity, while assuring a reasonable mean lesion-wise specificity, that in practice we chose to be 0.6. For this purpose, a two-threshold approach was employed: a first threshold is applied to generate candidate lesion areas in the image and a second threshold is employed to verify that at least one voxel of a candidate region is above this value. This process allows us to filter false positive regions. Threshold values equal to 0.00027 and

0.00134 were automatically selected using non uniform grid-searches to optimize the target objective on the validation data set. Finally a median filter of size 5 is applied on each sagittal slice to smooth noise mask boundaries. The resulting model achieved a lesion-wise sensitivity of 0.89 and a precision of 0.64 on the test set, so a relatively high sensitivity with precision close to its target value.

Model Implementation: The method was integrated into a pipeline developed in Python 3.8 and based on the following libraries: Pytorch-lightning (2.0.0), Torchio (0.18.87), PyTorch (1.13.1) and SimpleITK (2.2.1).

Supplementary Table 1 : MRI scanners characteristics

MRI constructors	*number of patients*	MRI models	*number of patients*
**Siemens**	**46**	**Area**	**25**
		**Prisma**	**16**
		**MAGNETOM Sola**	**2**
		**Skyra**	**2**
		**Avanto fit**	**1**
**Philips**	**2**	**Ingenia**	**2**
**General Electric**	**2**	**Signa Artist**	**1**
		**Optima MR450w**	**1**

Supplementary Table 2 : Lesion-wise sensitivity stratified by lesion volume (expressed in %, with 95% confidence intervals).

	**Overall lesion-wise sensitivity**		
	**readers without the tool**	**readers with the tool**	***p* value**
**Small lesions** (<100mm3)	59.6 (51.4-67.8)	64.0 (56.2-69.7)	0.00009
**Large lesions** (≥ 100 mm3)	78.2% (72.8-83.5)	81.4 (75.9-86.9)	0.039

Supplementary table 3: Classification of each MRI volume by readers with or without the tool, compared with the ground truth. GT = Ground truth

Without the tool	GT = 0 lesion	GT = 1 or 2 lesions	GT ≥ 3 lesions
reader = 0 lesion	463	81	19
reader = 1 or 2 lesions	164	509	124
reader ≥ 3 lesions	33	130	477
**With the tool**	**GT = 0 lesion**	**GT = 1 or 2 lesions**	**GT ≥ 3 lesions**
reader = 0 lesion	456	56	14
reader = 1 or 2 lesions	180	536	119
reader ≥ 3 lesions	24	128	487

Supplementary Figure 1: Web-based annotation tool used in the multi-reader study. A. Sagittal T2 (left) and sagittal STIR (right) cervical spinal cord acquisition without lesion mask generated by the automated tool. B. Sagittal T2 (left) and sagittal STIR (right) with lesion mask in blue generated by the automated tool for the same patient. C. Example of a lesion detected by the expert and by the automated tool (The expert's click on the lesion is visible in red)

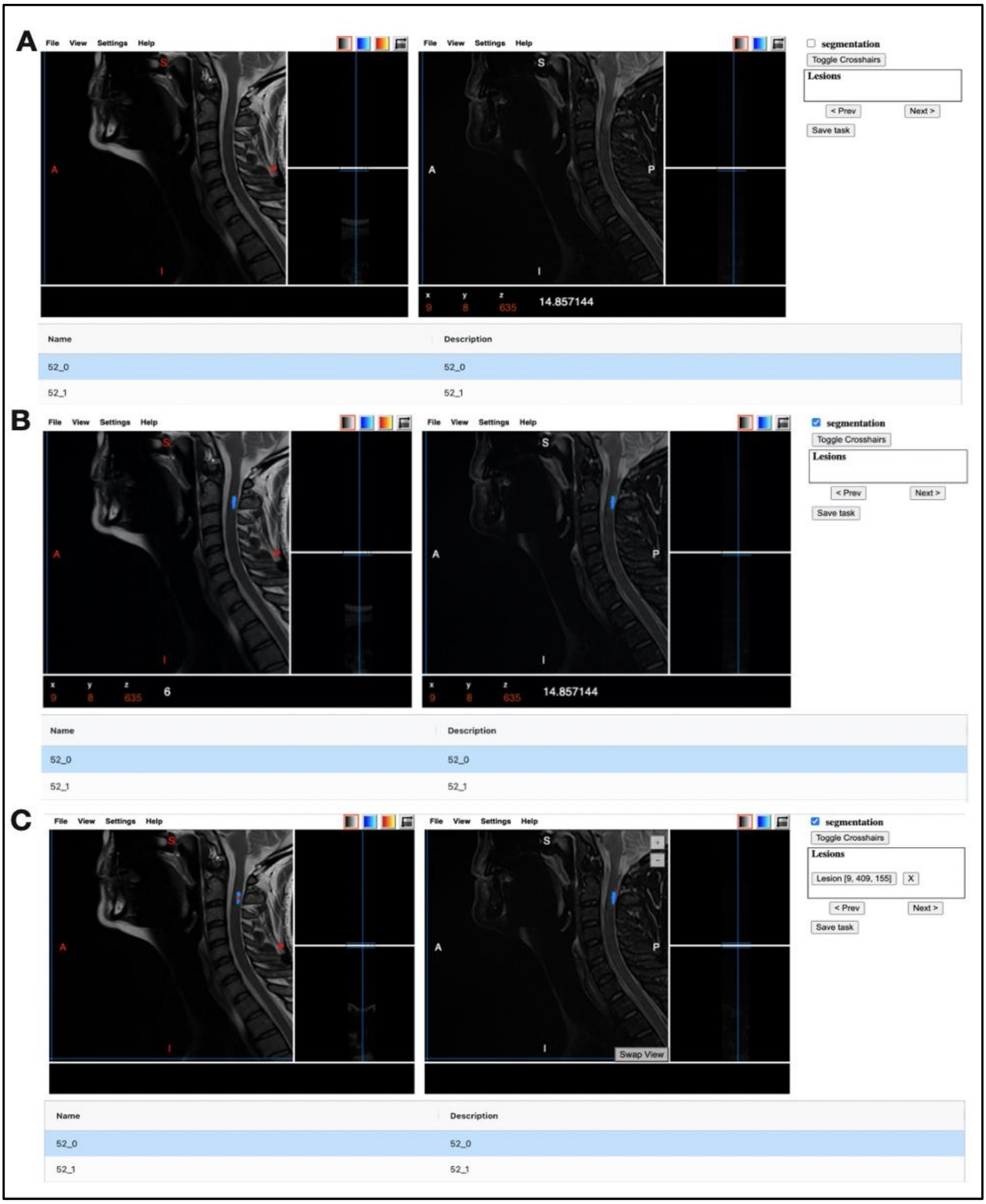

Supplement: Supplementary file 1 — ELECTRONIC SUPPLEMENTARY MATERIAL [file 330_2025_11541_MOESM1_ESM.pdf]
